# Radiogenomic modeling predicts survival-associated prognostic groups in glioblastoma

**DOI:** 10.1093/noajnl/vdab004

**Published:** 2021-02-15

**Authors:** Nicholas Nuechterlein, Beibin Li, Abdullah Feroze, Eric C Holland, Linda Shapiro, David Haynor, James Fink, Patrick J Cimino

**Affiliations:** 1 Paul G. Allen School of Computer Science & Engineering, University of Washington, Seattle, Washington, USA; 2 Department of Neurological Surgery, University of Washington, Seattle, Washington, USA; 3 Division of Human Biology, Fred Hutchinson Cancer Research Center, Seattle, Washington, USA; 4 Department of Radiology, University of Washington, Seattle, Washington, USA; 5 Department of Pathology, Division of Neuropathology, University of Washington, Seattle, Washington, USA

**Keywords:** biomarkers, copy number alterations, glioblastoma, MRI, radiogenomics

## Abstract

**Background:**

Combined whole-exome sequencing (WES) and somatic copy number alteration (SCNA) information can separate *isocitrate dehydrogenase (IDH)1/2*-wildtype glioblastoma into two prognostic molecular subtypes, which cannot be distinguished by epigenetic or clinical features. The potential for radiographic features to discriminate between these molecular subtypes has yet to be established.

**Methods:**

Radiologic features (*n* = 35 340) were extracted from 46 multisequence, pre-operative magnetic resonance imaging (MRI) scans of *IDH1/2*-wildtype glioblastoma patients from The Cancer Imaging Archive (TCIA), all of whom have corresponding WES/SCNA data. We developed a novel feature selection method that leverages the structure of extracted MRI features to mitigate the dimensionality challenge posed by the disparity between a large number of features and the limited patients in our cohort. Six traditional machine learning classifiers were trained to distinguish molecular subtypes using our feature selection method, which was compared to least absolute shrinkage and selection operator (LASSO) feature selection, recursive feature elimination, and variance thresholding.

**Results:**

We were able to classify glioblastomas into two prognostic subgroups with a cross-validated area under the curve score of 0.80 (±0.03) using ridge logistic regression on the 15-dimensional principle component analysis (PCA) embedding of the features selected by our novel feature selection method. An interrogation of the selected features suggested that features describing contours in the T2 signal abnormality region on the T2-weighted fluid-attenuated inversion recovery (FLAIR) MRI sequence may best distinguish these two groups from one another.

**Conclusions:**

We successfully trained a machine learning model that allows for relevant targeted feature extraction from standard MRI to accurately predict molecularly-defined risk-stratifying *IDH1/2*-wildtype glioblastoma patient groups.

Key Points

- Radiologic features separate risk-stratifying, WES/SCNA-defined glioblastoma subtypes.
- Tailored radiogenomic feature selection outperforms all-purpose feature selection methods.
- Contours on the T2-FLAIR MRI sequence may encode glioblastoma risk-stratifying information.

**Importance of the Study**

Whole exome sequencing and DNA somatic copy number alteration data are predictive of IDH-wildtype glioblastoma patient survival but are rarely clinically available. MRI is routinely available for glioblastoma patients, but its relationship to exome wide genomic data is unknown. In neuro-oncology, MRI-based radiogenomic models that associate imaging data with tumor genomic profiles are commonly sensitive to overfitting because MRI data is extremely high dimensional, and samples are limited. This study introduces a radiogenomic feature selection method that mitigates model overfitting, allows for the accurate prediction of survival-associated glioblastoma molecular profiles from MRI data, and indicates that features describing contours in the peritumoral edema and the infiltrating portions of glioblastoma visible on the T2-weighted FLAIR MRI sequence may differentiate these profiles. Because it is common for radiogenomic features to significantly outnumber the number of subjects in medical imaging studies, this method may be widely applicable.

Glioblastoma is a highly aggressive disease which is largely characterized by somatic copy number alterations (SCNAs), which are changes in chromosome structure resulting in gains or losses of either region of chromosomes (eg, *EGFR* amplification, *CDKN2A/B* deletion, and loss of chromosomal region 9p) or whole chromosomes (eg, gain of chromosome 7 and loss of chromosome 10).^1–5^ Certain SCNA signatures in glioblastoma have demonstrated prognostic utility with implications for risk-stratification beyond conventional histological grading.^3,6–9^ Using dimensionality reduction mapping of combined whole-exome single nucleotide mutations and exome-wide SCNAs, diffuse gliomas from The Cancer Genome Atlas (TCGA) display regional mapping clusters.^3^ Projection of a second independent cohort of paired initial and recurrent glioblastomas onto the TCGA reference map additionally indicates that there are genomic-based regions where patients have glioblastomas that are deemed unresectable at recurrence (Group 1) (Figure 1A). Furthermore, the *isocitrate dehydrogenase (IDH) 1* and *2* wildtype glioblastoma patients in this Group 1 have worse overall survival than those IDH-wildtype glioblastoma patients represented in the region overlapping with the paired cohort (Group 2) (Figure 1B). Group 1 tends to be relatively genomically stable with respect to copy number alterations beyond gain of chromosome 7, loss of chromosome 10, and loss of the chromosomal 9p region. We have previously shown that assignment to one of these two genomically determined groups (Figure 1A, C) is not predicted by clinical factors (age, sex, Karnofsky Performance Status) or epigenetic signatures (including genome-wide methylation and gene expression).^3^ The role of radiology in distinguishing these two groups is unknown. Given the implications of group membership to patient outcome, along with the relative abundance of pre-operative magnetic resonance imaging (MRI) and the lack of routine whole-exome sequencing of glioblastoma samples, we sought to determine whether radiographic features can differentiate Group 1 from Group 2. To do this, we turned to a cohort of IDH-wildtype glioblastomas with multisequence, pre-operative MRI in The Cancer Imaging Archive (TCIA) and used radiogenomic methods to train machine learning classifiers to discriminate between these two groups.

**Figure 1.**
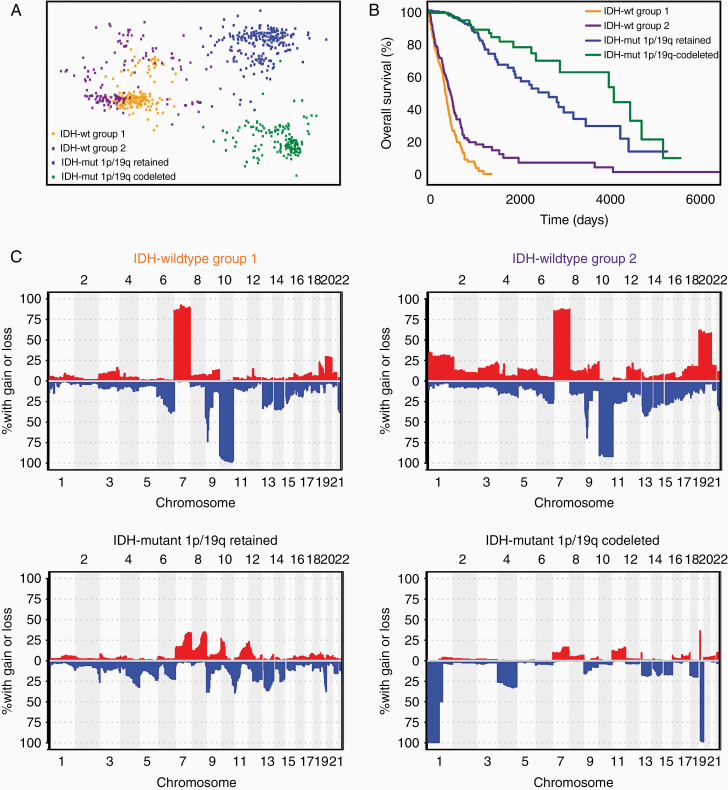
Characteristics of genomically defined prognostic diffuse glioma subtypes. (A) Multidimensional scaling plot of IDH-wildtype and IDH-mutant diffuse gliomas from the TCGA. Each point represents a single patient and is determined by dimensionality reduction from the combination of whole-exome sequencing and somatic copy number alterations. The more similar the genetic profile is between two patients, the closer their individual points are on the plot. The IDH-wildtype gliomas are divided into two previously determined prognostic subtypes (Groups 1 and 2). (B) Kaplan–Meier curves for IDH-wildtype (Groups 1 and 2) and IDH-mutant gliomas. (C) Copy number frequency plots of diffuse glioma subtypes showing the percentage of copy number alterations per molecular subtype (*y*-axis) over chromosome number and gene position (*x*-axis; for each chromosome moving from the distal end of the short *p* arm [left] to the distal end of the long *q* arm [right]).

Radiogenomics is an evolving field in medical imaging that employs supervised and unsupervised learning to relate quantitative imaging features to the underlying genomic characteristics of the imaged tissue.^10^ Radiogenomic pipelines typically consist of image acquisition, image normalization, feature extraction, and prediction using either coupled feature selection and machine learning models or end-to-end prediction using deep convolutional neural networks.^11,12^ In neuro-oncology, radiogenomic approaches have been used to predict IDH mutations,^12–16^ 1p/19q-codeletion,^16,17^  *O6-methylguanine-DNA methyltransferase* promoter methylation,^17,18^ several gene-level SCNAs,^19^ and tumor treatment responses.^20^ Though powerful, radiogenomic methods in neuro-oncology are prone to overfit classification tasks because MRI data is high dimensional, relatively scarce, extremely heterogenous, and inherently noisy because of MRI scanner manufacturer, sequence protocol, and patient movement vary. This is especially true for complex machine learning models, such as deep neural networks, which excel in certain glioma imaging tasks, such as tumor segmentation, but struggle with nuanced classification and regression tasks, such as survival prediction.^21,22^ Even traditional machine learning models can be overwhelmed by the size of the feature spaces extracted by open-source radiogenomic software (*n* > 10 000).^23,24^ To reduce dimensionality, standard feature selection methods such as least absolute shrinkage and selection operator (LASSO) feature selection, recursive feature elimination, and variance thresholding are commonly employed. However, these methods can also overfit classification tasks in small studies where predictions must be made on large, noisy data. To address this, we propose a novel feature selection method that allows simple machine learning models to accurately predict Groups 1 and 2 and gives insight into what radiographic features discriminate these two molecularly defined groups from one another.

## Materials and Methods

### TCGA Glioma Datasets

Genomic whole-exome sequencing and somatic copy number alteration data for TCGA glioblastomas, as well as lower-grade astrocytic and oligodendroglial tumors, were obtained from the University of California Santa Cruz cancer browser (https://genome-cancer.ucsc.edu/) as previously described.^3,4^ From the whole-exome sequencing data, we incorporated nonsynonymous nucleotide point mutations into our analysis as previously described.^3–5^ For copy number alterations, single nucleotide polymorphism (SNP)-array derived GISTIC 2.0 scores were used for analysis.^3–5^ Dimensionality reduction and visualization of the exome-wide point mutations combined with copy number alterations were performed for each patient using multidimensional scaling (MDS) as previously described.^3–5^ Diffuse gliomas were binned into three broad categories: IDH-wildtype, IDH-mutant, and 1p/19q retained, and IDH-mutant and 1p/19q-codeleted. IDH-wildtype diffuse astrocytic gliomas were further divided into two prognostic groups, which we label Groups 1 and 2 (Supplementary Table 1), based upon previously published MDS and mapping of an independent cohort of paired initial and recurrent glioblastomas.^3^ Of these IDH-wildtype gliomas, we considered only the subset of World Health Organization (WHO) grade IV glioblastomas.

### The Cancer Imaging Archive Magnetic Resonance Imaging

Multisequence MRI volumes for patients with IDH-wildtype glioblastoma were downloaded from TCIA data portal (https://public.cancerimagingarchive.net/nbia-search/).^25^ Of these patients, 46 met the inclusion criteria requiring available SCNA and whole-exome sequencing (WES) data from the TCGA and usable pre-operative pre- (T1) and postcontrast (T1ce) T1-weighted sequences along with T2-weighted (T2) and T2 Fluid-Attenuated Inversion Recovery (FLAIR) sequences (scanner details listed in Supplementary Table 2).^26^ Group 1 consisted of 25 patients (median age 59 years; 10 female, and 15 male); Group 2 consisted of 21 patients (median age 63 years; 9 female, and 12 male). The Brain Extraction Tool (BET) and FMRIB’s Linear Image Registration Tool (FLIRT) from the FMRIB Software Library (FSL) were used to skull-strip and co-register same-subject MRI sequences.^27,28^ All MRI volumes were resampled to 1 mm^3^ isotropic space. Each tumor was automatically segmented into subcompartments using a publicly available segmentation model^29^ pretrained on the 2018 Multimodal Brain Tumor Segmentation Challenge (BraTS) MRI dataset.^21,26,30^ This model placed each tumor voxel into one of three tumor regions used in the BraTS challenges’ segmentation scheme: the contrast-enhancing tumor compartment, the nonenhancing tumor and necrotic tissue, and the peritumoral edema tumor region. We modified our predicted segmentation maps to conform to a different segmentation scheme we regard as more intuitive (Supplementary Figure 1). We maintained the same contrast-enhancing tumor compartment definition as the BraTS challenges, but we split the nonenhancing tumor and necrotic tissue regions. We considered necrotic tissue as a tumor region on its own, and we merged the nonenhancing tumor region with the BraTS challenges’ peritumoral edema tumor region to form a tumor region referred to as the T2 abnormality. In our view, nonenhancing tumor tissue is challenging to distinguish from peritumoral edema, which must be done in the BraTS segmentation scheme. In our scheme, however, bright contrast-enhancing tumor and dark necrotic tissue are easily identifiable on the T1ce sequence; all other tissue appearing abnormal on the T2 or FLAIR sequence is, by definition, the T2 abnormality tumor region. In addition to the enhancing tumor region, necrotic tissue, and the T2 abnormality, we also considered the tumor core, formed by merging the enhancing and necrotic regions, and the entire tumor as additional tumor regions. All segmentation masks were examined and manually corrected using Insight Segmentation and Registration Toolkit (ITK-SNAP) to the satisfaction of at least one experienced neuro-radiologist at The University of Washington Medical Center (JF and DH).^31^ Finally, N4 bias field correction and min-max normalization were applied to the MRI data.^32^

### Statistical Methods

We trained and evaluated six classes of machine learning models including LASSO models, linear support vector machines (SVM), multilayer perceptrons (MLP), XGBoost models, random forest models (RF), and ridge logistic regression (LR) classifiers on features selected by our proposed feature selection method (Python, Version 3.7, www.python.org). Model performance was measured by the area under the receiver operating characteristic curve (AUC). All reported numbers are the average AUC of 100 trials of 10-fold cross-validation run on randomly chosen partitions. Receiver operating characteristic (ROC) curves were also used to compare model performances. Feature selection, principle component analysis (PCA) dimensionality reduction, and model evaluation were conducted inside of the cross-validation loop. To avoid overfitting, we used the default model hyperparameters from Python’s *scikit-learn* and *xgboost* packages.^33,34^ All reported selected features were identified by conducting feature selection on the entire dataset after all parameters had been chosen.

## Results

### MRI Feature Extraction

**Figure 2.**
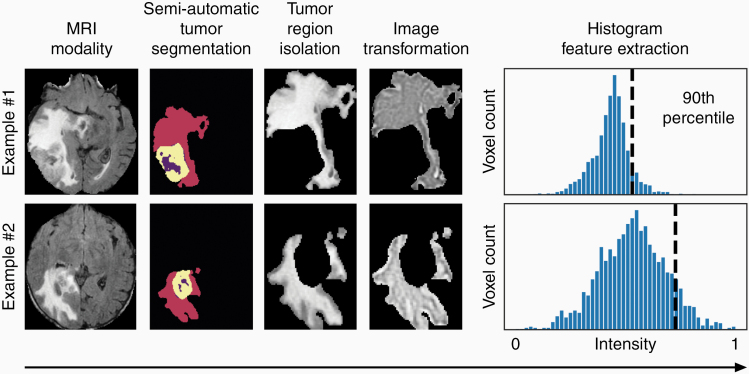
Diagram of radiomic feature extraction from MRI. Feature extraction begins by categorizing the MRI sequence (FLAIR imaging shown in these examples). The sequence images then undergo semi-automatic tumor segmentation followed by tumor region isolation. The isolated tumor region then undergoes an image transformation (Laplacian of Gaussian transformation shown in these examples). Here, a histogram feature is derived from the intensity values of the tumor regions and the nth percentile value. This defines an extracted feature (90th percentile shown in these examples).

To build a prediction model based upon radiographic signatures, we first represented each patient by a set of quantitative MRI features. To do this, we extracted semantically interpretable histogram and texture features from augmented volumes of interest (VOIs) formed by the application of an image transformation to a tumor region on each MRI sequence. We formed 380 VOIs by independently transforming the 5 tumor regions in each of the 4 MRI sequences with 19 stationary image filters (Supplementary Tables 3–5). A total of 35 340 radiographic histogram/texture features (Supplementary Table 6) were extracted per patient using 93 histogram and texture features (Supplementary Table 7). Our feature extraction pipeline is illustrated in Figure 2. Texture features consist of formulas that describe the distribution of values on second-order texture matrices such as the grey level co-occurrence matrix (GLCM).^35^ Examples of image transformations include local binary patterns (LBP), Laplacian of Gaussian (LoG) filters, and Haar wavelet transformations.^36,37^ Sixty shape features that describe the VOIs, such as tumor region volume and surface area, and 16 Visually AcceSAble Rembrandt Images (VASARI) MRI features,^38^ which are manually extracted features designed to standardize visual descriptions of gliomas, were also extracted to ascertain whether visually perceptible tumor characteristics can distinguish Group 1 from Group 2 (Supplementary Tables 8 and 9).

### MRI Feature Selection

**Figure 3.**
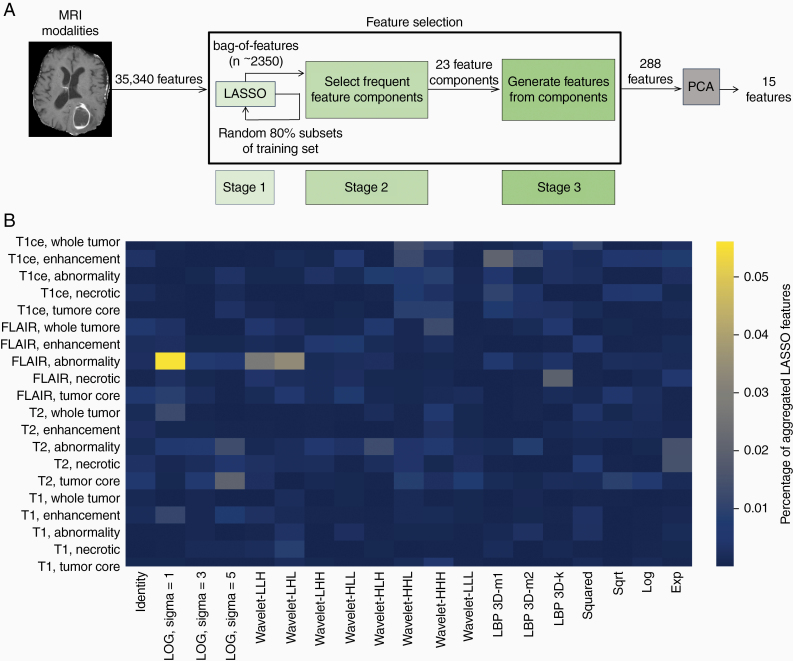
MRI feature selection schematic. (A) Feature selection pipeline starting with MRI extracted features and ending with 288 radiogenomic features. PCA further reduces the feature set to 15 principal components that are used to train machine learning models to predict patients’ placement into IDH-wildtype molecular Group 1 versus Group 2. (B) Heatmap example of radiographic features selected in the first stage of our feature selection method. This shows the distribution of the features aggregated in the bag-of-features *B* over VOIs defined by a combination of MRI sequence, tumor region, and image transformation. Notably, the VOI defined by the FLAIR sequence, T2 abnormality, and LoG image transformation with kernel width 1 mm is the most common source of radiographic features in *B*.

Following the feature extraction process, we sought to identify the subset of features that are most important to the prediction task. We developed a feature selection method that prioritized identifying characteristics of radiographic features—including MRI sequence, tumor region, image transformation, and histogram/texture formulas—rather than specific radiographic features. We refer to these characteristics as feature components. Because VOIs are each defined by a combination of feature components, our strategy amounts to selecting a set of VOIs and a set of texture/feature formulas that can extract numeric radiogenomic features from these VOIs. The development of our method was guided by our expectation that discriminative radiogenomic features were contained in only a few feature component-defined VOIs and captured by only a few histogram/texture formulas. To select a subset of feature components, we trained LASSO models on random subsets of the training data and used the features they selected to aggregate a bag of repeatedly selected radiographic features. We then broke down the radiographic features in this aggregated bag into their feature components and selected the MRI sequences, tumor regions, image transformations, and histogram/texture formulas that appeared most frequently. Finally, from this set of selected feature components, we identified the set of radiographic features defined by the selected MRI sequences, tumor regions, image transformations, and histogram/texture formulas we selected. We trained our ultimate classification models on a 15-dimensional embedding of this feature set produced by PCA. Our feature selection process is summarized in Figure 3.

Formally, in the first stage of our feature selection method, we aggregated a bag *B* of LASSO-selected features, including duplicates, by training 50 LASSO models on random subsets of 80% of the training data. The size of this bag varied slightly depending on which subsets were chosen (*n* = 2350 ± 80). In the second stage, we used *B* to determine which feature components were most relevant to the classification task. This was done by examining the distribution of *B* over each feature component category. From these distributions, we selected a set *C* of the most frequently appearing 3 tumor regions, 3 MRI sequences, 4 image transformations, and 8 histogram/texture formulas, where thresholds were determined empirically (Supplementary Table 10). In the third stage, we generated the set of 288 features (Figure 3; Supplementary Table 11) whose components were determined from the set *C* by selecting those radiographic features whose feature components were all contained in *C*. Finally, we used PCA to further reduce the dimensionality of our feature set to 15.

The most interpretable selected histogram/texture formulas include histogram skewness and kurtosis and GLCM cluster shade, contrast, and informational measure of correlation. These features describe the symmetry and peakiness of an image-transformed VOI’s intensity histogram and the uniformity, variation, and row-column correlation of the values in its grey-level co-occurrence matrix. Histogram symmetry and peakiness, as well as texture uniformity, may describe the diffusivity of gliomas which is linked to prognosis. The T1ce, FLAIR, and T2 MRI sequences were selected in the second stage of our feature selection method, as were the T2 abnormality, whole tumor, and tumor core tumor regions, and two Laplacian of the Gaussian (LoG) and two Haar wavelet image transformations. The VOI defined by the FLAIR sequence, T2 abnormality region, and LoG image transformation with kernel width 1mm is likely the primary source of radiographic features that contribute most to the classification tasks because these feature components were disproportionately represented in the bag *B* of aggregated features produced in the first stage of our feature selection method (Figure 3B). Moreover, because the LoG image transformation is an edge detector, we posit that the best discriminating MRI features describe the contours of hyperintensity within the transformed T2 abnormality region derived from the FLAIR sequence.

### Radiogenomic Model Prediction

To evaluate the efficacy of our feature selection method, we compared the results of machine learning models trained to bin patients into respective IDH-wildtype genomic groups with our feature selection method to the results of similar models trained with the following standard feature selection methods: LASSO feature selection, recursive feature elimination, and variance thresholding. For fair comparison across models, all feature selection methods were forced to select exactly 288 features and were evaluated with the application of 15-dimensional PCA reduction. Additionally, to determine the utility of our feature selection method beyond reducing the dimension of model input, we compared our results to those of models trained on the 15-dimensional PCA embedding all 35 340 extracted radiographic features (Supplementary Figures 2–5). Further, to better understand the extent to which molecular Group 1 and molecular Group 2 can be distinguished visually, we trained models on 60 tumor shape features and 16 VASARI MRI features for comparison.

**Figure 4.**
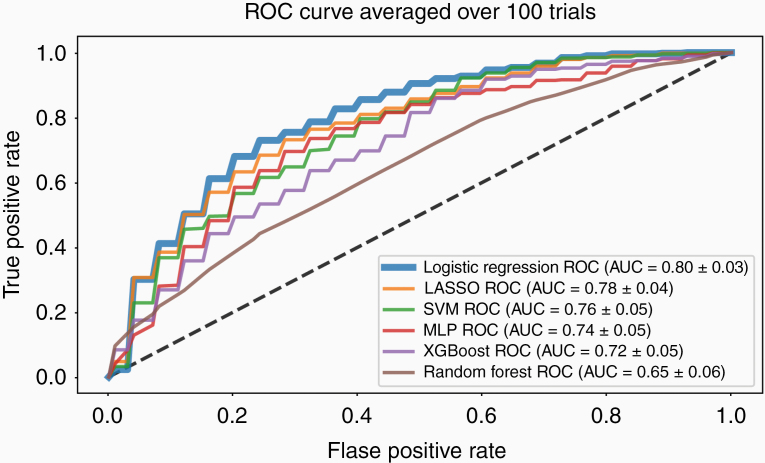
Comparison of ROC curves for various machine learning models that predict assignment to Group 1 versus Group 2 from MRI.

**Figure 5.**
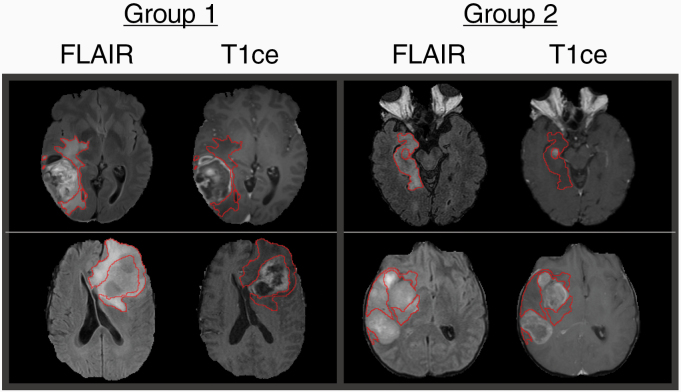
Examples of MRI volumes consistently correctly classified by ridge logistic regression. The T2 abnormality region boundary is outlined in red on the FLAIR and postcontrast T1ce images. Our results indicate that the T2 abnormality on the FLAIR sequence best discriminates between molecular Group 1 and molecular Group 2; however, this is not obvious to visual inspection. Sharply defined T2 abnormality region boundaries may be more apparent in Group 1, and more infiltrative T2 abnormality regions may be more common in Group 2.

IDH-wildtype glioblastoma molecular groups 1 and 2 were classified with a cross-validated AUC score of 0.80 (±0.03) using a ridge logistic regression model trained with our PCA-reduced feature selection method (Figure 4). All models that were trained with our method significantly outperformed the same models trained with LASSO feature selection, recursive feature elimination, and variance thresholding (Table 1). Moreover, models trained with our feature selection method outperformed models trained on the 15-dimensional PCA embedding of all 35 340 features (Table 1). This comparison controls for model input dimension and indicates that the improvement in performance provided by our feature selection method was due to selecting richer features rather than simply reducing the number of input features. Unlike models trained with our feature selection method, models trained with the standard feature selection methods we evaluated underperformed those trained on the PCA embedding of all extracted features. This supports the notion that our method is able to mitigate the risk of overfitting which is more common in other feature selection methods. The fact that no model trained on shape or a VASARI features achieved an AUC score over 0.6 is a testament to the difficultly of distinguishing molecular Group 1 from molecular Group 2 with straightforward visual features. Examples of MRI volumes that were consistently correctly classified across cross-validation folds are shown in Figure 5 to showcase this difficulty and point out possible patterns.

**Table 1.** Cross-Validated AUC Results of 6 Machine Learning Modeled Trained on the Indicated Input Features

Input Features	LASSO	SVM	MLP	XGBoost	RF	LR
Ours	0.78	0.76	0.74	0.72	0.65	0.80
LASSO feature selection	0.59	0.58	0.63	0.56	0.54	0.61
Recursive feature elimination	0.58	0.58	0.61	0.55	0.54	0.59
Variance thresholding	0.63	0.61	0.65	0.63	0.62	0.68
All features (PCA = 15)	0.72	0.67	0.68	0.64	0.71	0.74
All features	0.59	0.69	0.69	0.7	0.66	0.76
VASARI features	0.42	0.46	0.41	0.59	0.48	0.43
Shape features	0.53	0.36	0.53	0.4	0.49	0.51

All numbers are the average AUC of 100 trials of 10-fold cross-validation run on randomly chosen partitions.

We evaluated models and feature selection methods across a range of PCA dimension choices and chose the lowest round number (*n* = 15) above which most models’ performance plateaued (Supplementary Figures 2–5). Importantly, models trained with our feature selection method performed best across the range of choices of PCA dimension we evaluated (3–40), as well as when PCA was not applied (Supplementary Figure 2–6, Supplementary Table 12). Interestingly, the results produced by models trained on feature selection methods without the application of PCA were mostly similar to those trained with PCA (Supplementary Table 12). Of note, our method was slightly more effective without the use of PCA (AUC = 0.82 ± 0.03). Other changes induced by the application of PCA were decreased performances of random forest and XGBoost models trained on LASSO and recursive feature elimination, which was significantly higher when PCA was not applied, though their AUC scores did not exceed 0.70. These models also performed well when LASSO feature selection was permitted to select fewer than 288 features: random forest and XGBoost models achieved AUC scores of 0.76 (±0.06) and 0.78 (±0.07), respectively, when trained on small (*n* < 10) sets of features selected by LASSO (Supplementary Figure 3). Though these results were promising, the performance was unstable and the features LASSO selected varied greatly between cross-validation folds, hindering interpretability. In general, models trained with LASSO feature selection, recursive feature elimination, and variance thresholding methods, even when there were not constrained to select 288 features, performed worse than models trained with our feature selection method (Supplementary Figures 3–5).

## Discussion

We developed a novel LASSO-based feature selection method that improved the ability of machine learning classification algorithms to delineate prognostic molecular subgroups of IDH-wildtype glioblastomas originally defined by combined WES and SCNAs. In particular, a ridge logistic regression classifier trained on a 15-dimenisonal PCA embedding of 288 selected radiogenomic features predicted these two groups with cross-validated AUC = 0.80 (±0.03). We additionally conjectured that the T2 abnormality tumor region in the FLAIR MRI sequence under a Laplacian of Gaussian (LoG) edge detector image transformation may be the primary source of the most discriminative signal in this task. We suspect this for the following two reasons. First, the T2 abnormality is the most prominent tumor region on the FLAIR sequence and both the T2 abnormality and FLAIR sequence were selected in the second stage of our feature selection method (Supplementary Table 10). Second, VOIs constructed from the FLAIR sequence and T2 abnormality were the most common source of features selected in the first stage of our method (Figure 3B). If true, fine-grain contours in the peritumoral edema and the infiltrating portion of glioblastomas on the FLAIR MRI sequence may be the basis for a characterization of the difference between molecular Groups 1 and 2. However, this difference may be difficult for the human eye to parse. Were pronounced differences in FLAIR contours and infiltration visible, we would expect VASARI features to be better discriminators of Groups 1 and 2 than we observed. Similarly, while examinations of correctly classified MRI samples may suggest that T2 abnormality region boundaries may be better defined in Group 1 and appear more infiltrative in Group 2, these observations are neither obvious nor conclusive (Figure 5). Additionally, the absence of the identity image transformation in the set of selected feature components may also attest to the difficulty of distinguishing between Group 1 and 2 by eye. We are more confident that tumor enhancement does not separate these groups. While our method selected the T1ce sequence, it did not select the enhancing tumor region, likely because the presence of tumor enhancement on the T1ce sequence of IDH-wildtype glioblastoma MRI scans is nearly ubiquitous and thus may not differ significantly between molecular Groups 1 and 2 (Supplementary Table 10).

Our cross-validated results showed that models trained with our feature selection method outperformed baseline models trained without feature selection on the same 35 340-dimensional datasets and its 15-dimensional PCA embedding. On the other hand, models trained with LASSO feature selection, recursive feature elimination, and variance thresholding benchmarked well below these baseline results, a clear sign of overfitting. This indicates that our method is more robust to overfitting than three standard feature selection methods on a small set of high dimensional data. The observation that linear models, such as logistic regression and LASSO, outperformed more complex models, such as random forests and support vector machines, shows the benefit of using simple models in such settings to avoid overfitting in the postfeature selection modeling phase.

This study’s contribution to radiogenomics is a multistage feature selection method that boosted the performance of a set of machine learning classifiers in a situation where the number of features significantly outnumbered the number of training samples. Such situations are likely to remain commonplace in medical imaging as the accrual of pre-operative MRI data is constrained by the limited cases of glioblastoma.^39^ Further, with the inception of open source radiogenomic feature extraction software, such as *pyradiomics* and the Cancer Imaging Phenomics Toolkit (CaPTk), the MRI sequence, tumor region, and imaging transformation-based radiogenomic feature structure that our method leverages is increasingly becoming the standard, thereby ensuring the relevance of our method.

Moving forward, additional cohorts will be necessary to rigorously validate our method. However, currently there is a lack of available datasets for patients with de novo IDH-wildtype glioblastomas that contain WES, SCNA data, and multiple MRI sequences. Nonetheless, if our method’s robustness is further supported outside of our current cross-validation study, such an MRI-based classifier capable of distinguishing poor surviving Group 1 IDH-wildtype glioblastoma patients from longer surviving Group 2 IDH-wildtype glioblastoma patients could have an immediate influence on patient care. Shorter-term Group 1 patients, who are also less likely to have surgical intervention for tumor recurrence,^3^ could be recommended for upfront clinical trials. Within trials, if Groups 1 and 2 patients are not balanced in phase II and phase III arms, incorrect and costly conclusions may be drawn. Inferring patient risk stratification from available baseline data such as MRI is a critical way forward in neuro-oncology.

## Supplementary Material

vdab004_suppl_Supplementary_Figures

vdab004_suppl_Supplementary_Tables

## Notes

**Authorship Statement.** N.N. and P.J.C. designed the study. Data collection was performed by N.N., B.L., A.F., D.H., and J.F. analysis and interpretation of the data were done by N.N., E.C.H., L.S., D.H., J.F., and P.J.C. The manuscript was drafted by N.N. and P.J.C. All authors participated in revising and approval the manuscript.
