# Supplementary material for: Radiogenomic modeling predicts survival-associated prognostic groups in glioblastoma
Source: Neurooncol Adv. 2021 Feb 15;3(1):vdab004. doi: 10.1093/noajnl/vdab004 (PMC7883769; doi:10.1093/noajnl/vdab004)

SUPPLEMENTARY MATERIAL

Supplementary Figure 1. Comparison of segmentation schemes. Two T1ce MRI Modalities with segmentation labels overlaid. On the right, the Multimodal Brain Tumor Segmentation Challenges (BraTS) segmentation scheme. On the left, we show our scheme. Notably, we merge the BraTS challenges' non-enhancing tumor and edema regions and refer to the merged region as the T2 abnormality.

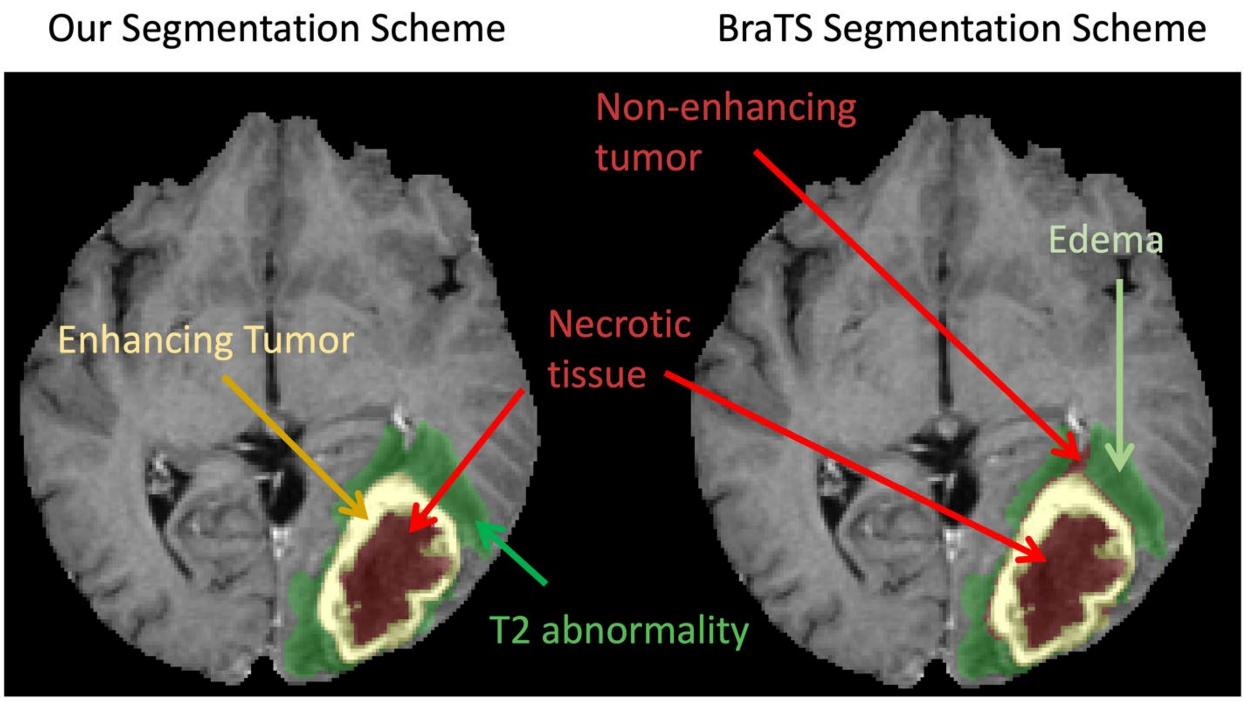

Supplementary Figure 2. Evaluation of models trained with our feature selection method with varying choices of PCA dimension. We plot PCA dimension less than or equal to the number of samples used in each cross-validation fold (n=40).

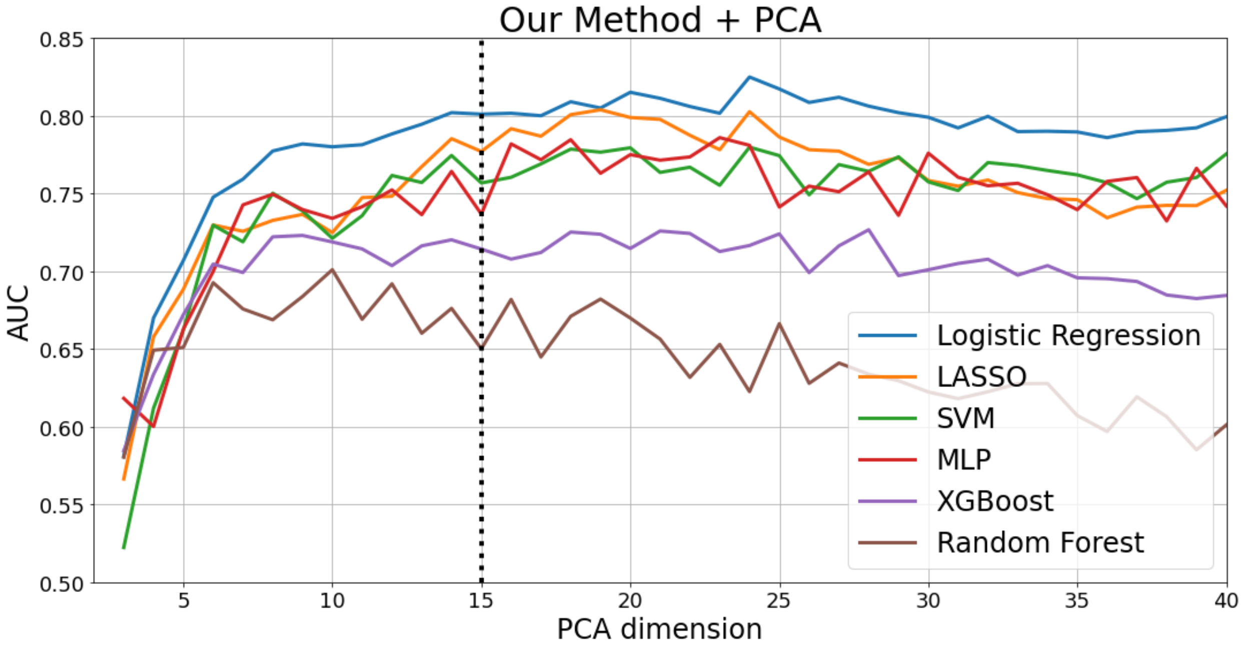

Supplementary Figure 3. Performance of models trained using LASSO feature selection. Random forest and XGBoost models do perform well (AUC=0.76, 0.78) when trained on small sets of features selected by LASSO, but the features LASSO selects vary greatly between cross-validation folds which renders the models unreliable and difficult to interpret. Model without (A) and with (B) incorporation of PCA.

A)

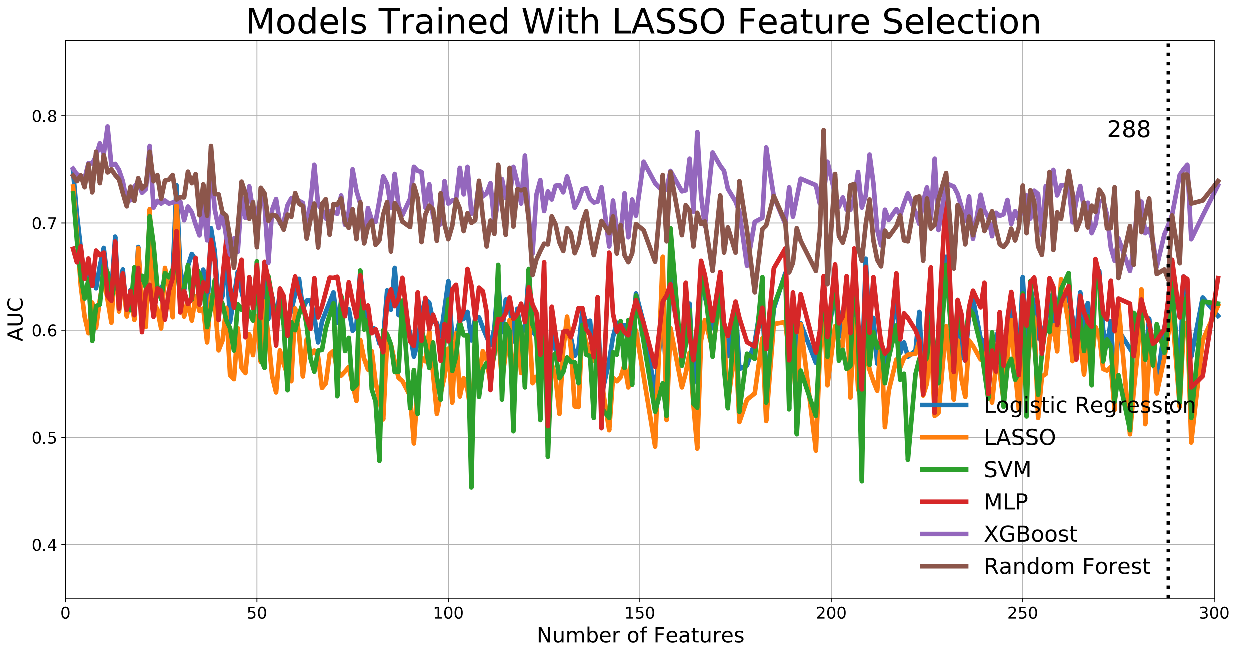

B)

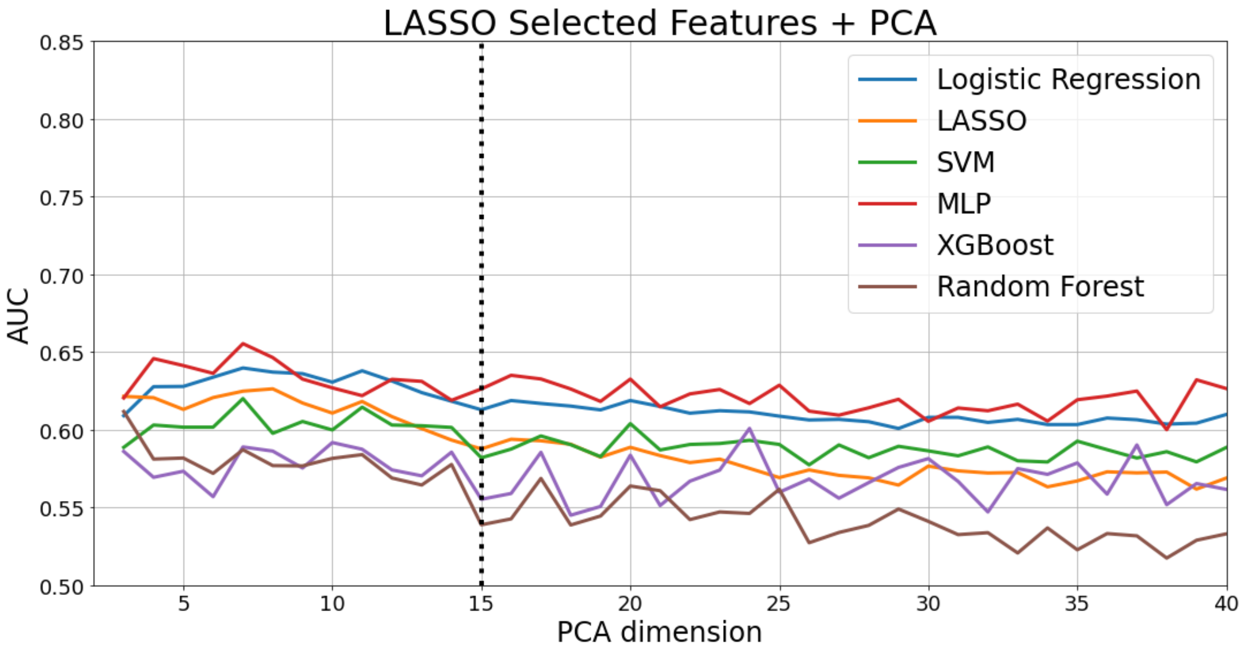

Supplementary Figure 4. Performance of models trained using recursive feature elimination selection. Model without (A) and with (B) incorporation of PCA.

A)

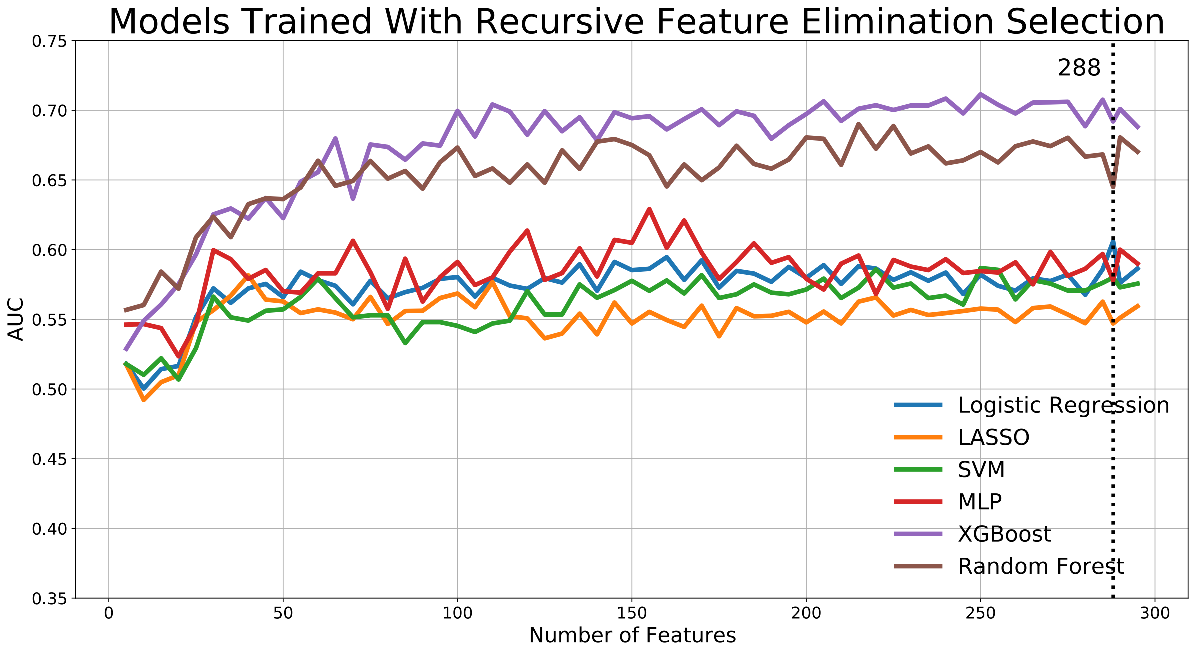

B)

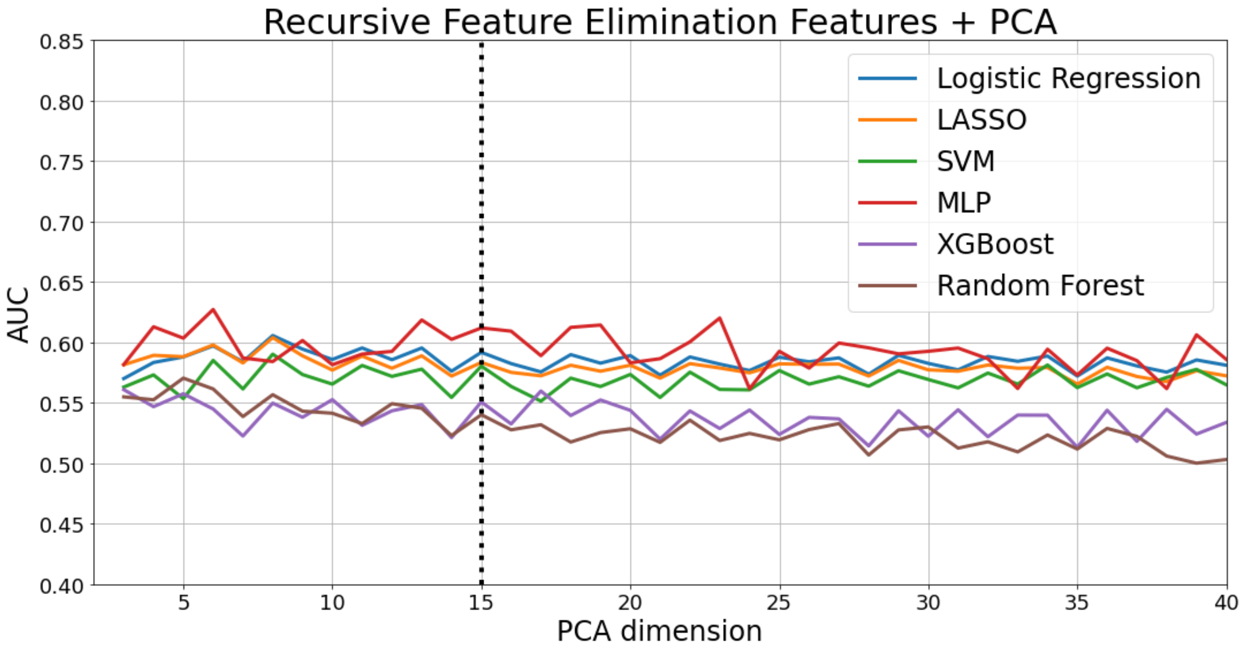

Supplementary Figure 5. Performance of models trained using variance thresholding feature selection. Model without (A) and with (B) incorporation of PCA.

A)

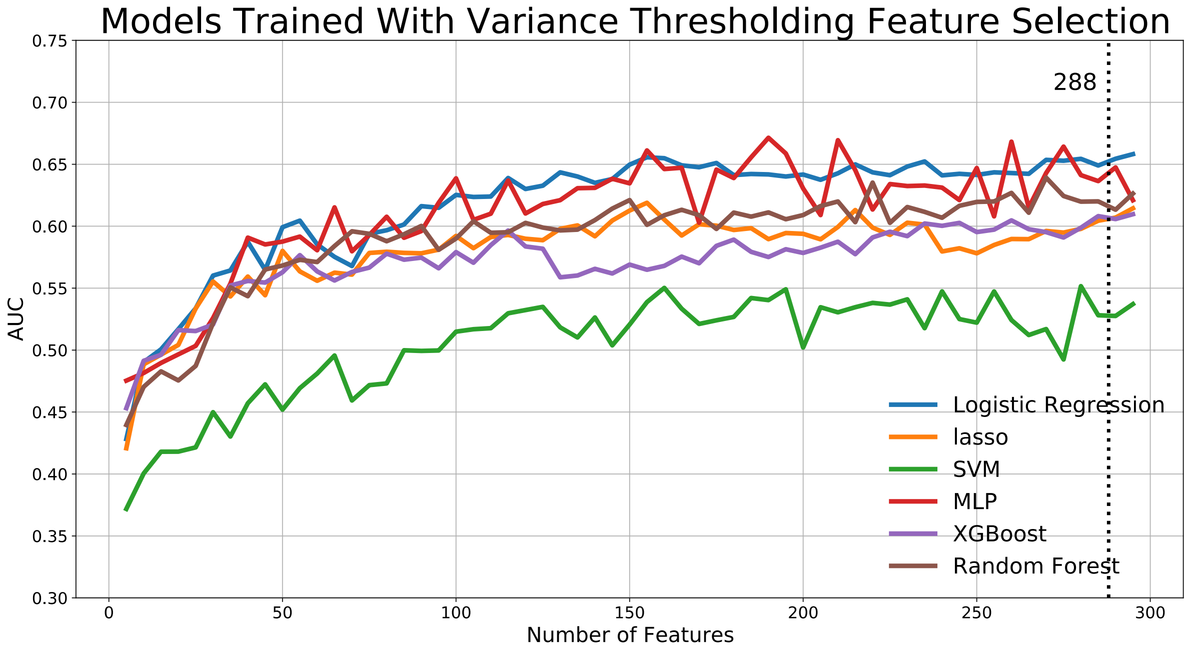

B)

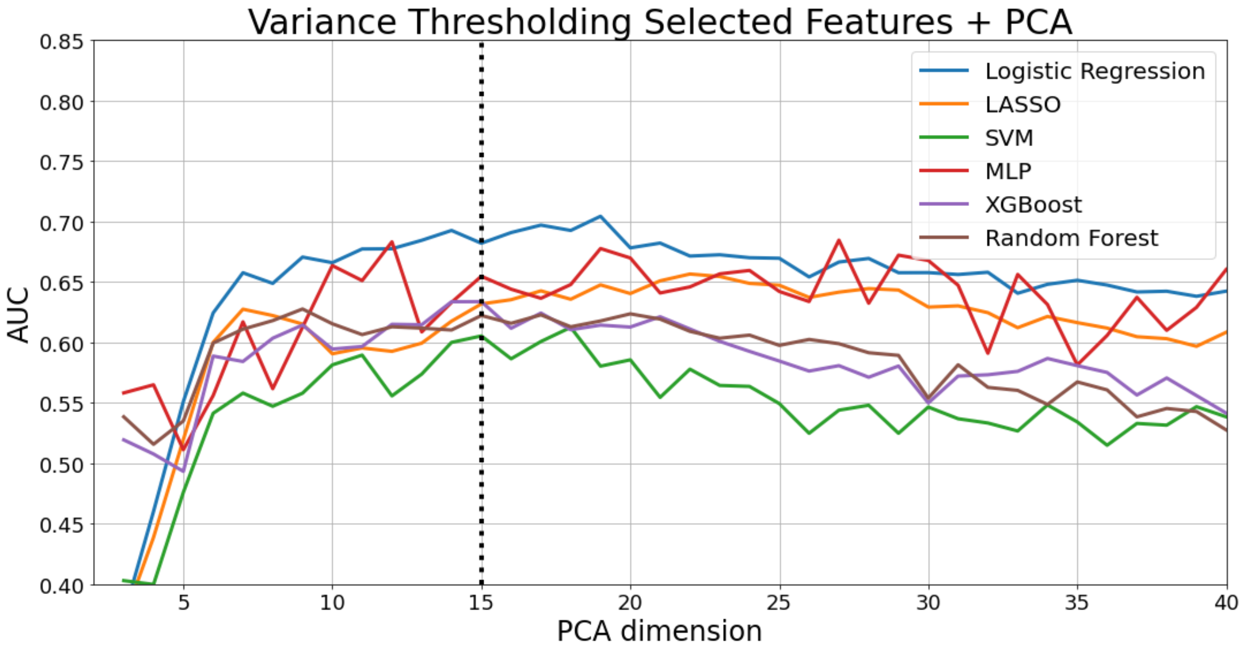

Supplementary Figure 6. Performance of models trained on PCA embeddings of all features (without feature selection) at various PCA dimensions.

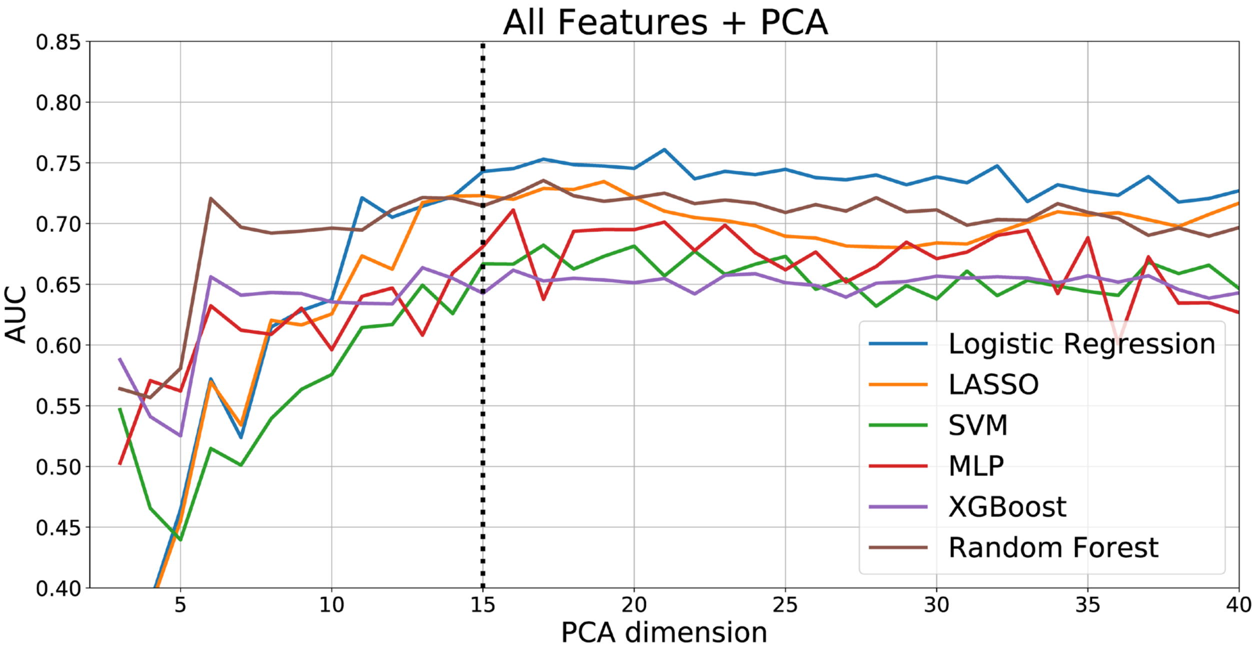

Supplement: vdab004_suppl_Supplementary_Figures [file vdab004_suppl_supplementary_figures.pdf]
